# Using SWAT to Evaluate Streamflow and Lake Sediment Loading in the Xinjiang River Basin with Limited Data

**DOI:** 10.3390/w12010039

**Published:** 2019-12-20

**Authors:** Lifeng Yuan, Kenneth J. Forshay

**Affiliations:** 1 National Research Council Resident Research Associate at the United States Environmental Protection Agency, Robert S. Kerr Environmental Research Center, 919 Kerr Research Drive, Ada, OK 74820, USA; 2 U.S. Environmental Protection Agency, Center for Environmental Solutions and Emergency Response, Robert S. Kerr Environmental Research Center, 919 Kerr Research Dr., Ada, OK 74820, USA

**Keywords:** SWAT, SWAT-CUP, soil erosion, streamflow modeling, sediment yield, Xinjiang River basin

## Abstract

Soil erosion and lake sediment loading are primary concerns of watershed managers around the world. In the Xinjiang River Basin of China, severe soil erosion occurs primarily during monsoon periods, resulting in sediment flow into Poyang Lake and subsequently causing lake water quality deterioration. Here, we identified high-risk soil erosion areas and conditions that drive sediment yield in a watershed system with limited available data to guide localized soil erosion control measures intended to support reduced sediment load into Poyang Lake. We used the Soil and Water Assessment Tool (SWAT) model to simulate monthly and annual sediment yield based on a calibrated SWAT streamflow model, identified where sediment originated, and determined what geographic factors drove the loading within the watershed. We applied monthly and daily streamflow discharge (1985–2009) and monthly suspended sediment load data (1985–2001) to Meigang station to conduct parameter sensitivity analysis, calibration, validation, and uncertainty analysis of the model. The coefficient of determination (*R*^2^), Nash–Sutcliffe efficiency (*NSE)*, percent bias (*PBIAS*), and RMSE -observation’s standard deviation ratio (*RSR*) values of the monthly sediment load were 0.63, 0.62, 3.8%, and 0.61 during calibration, respectively. Spatially, the annual sediment yield rate ranged from 3 ton ha^−1^year^−1^ on riparian lowlands of the Xinjiang main channel to 33 ton ha^−1^year^−1^ on mountain highlands, with a basin-wide mean of 19 ton ha^−1^year^−1^. The study showed that 99.9% of the total land area suffered soil loss (greater than 5 ton ha^−1^year^−1^). More sediment originated from the southern mountain highlands than from the northern mountain highlands of the Xinjiang river channel. These results suggest that specific land use types and geographic conditions can be identified as hotspots of sediment source with relatively scarce data; in this case, orchards, barren lands, and mountain highlands with slopes greater than 25° were the primary sediment source areas. This study developed a reliable, physically-based streamflow model and illustrates critical source areas and conditions that influence sediment yield.

## 1. Introduction

Soil erosion and lake sediment loading are severe ecological and environmental problems that watershed managers face around the world. In subtropical Southeastern China, both climatological and anthropogenic activity have altered hydrology and sediment loading [1]. In these areas, concentrated rainstorms occur during the monsoon period (May–October), where intense regional precipitation likely drives much of the soil erosion [2,3]. Erosive rainfall strips valuable topsoil away and, subsequently, sediment flows into nearby streams or water bodies, ultimately contributing to land degradation and downstream contamination, including nonpoint source pollution, siltation of reservoirs and lakes, and further deterioration of water quality [4]. Land use change is often identified as a manageable primary factor in soil erosion [4]. To address these erosion problems, managers often rely on modeling tools, but limited data availability discourages the application of many of the most accepted tools due to concerns regarding calibration and validation, which can prevent determination for focused land use management. For example, many physically-based hydrological models have been developed, such as the Areal NonPoint Source Watershed Environmental Response Simulation (ANSWERS) [5], the Agricultural Nonpoint Source Pollution Model (AGNPS) [6], the Better Assessment Science Integration Point and Nonpoint Sources (BASINS) [7], the Hydrologic Simulation Program Fortran (HSPF) [8], the Simulator for Water Resources in Rural Basins (SWRRB) [9], the Water Erosion Prediction Project (WEPP) [10], and the Soil and Water Assessment Model (SWAT) [11], among others, which are used widely to estimate streamflow processes, simulate sediment yield and transport, identify soil erosion high-risk areas, evaluate nonpoint source pollution, support water quality criteria (e.g., total maximum daily loads (TMDLs)) development, and support decision-making at the local or regional levels [12–15]. These are generally data intensive models [16]. Among those models, SWAT has been applied across various spatial and temporal scales and environmental conditions worldwide as a common watershed analysis tool [17]. Due to its distributed, physically-based structure, SWAT needs many input data to meet the requirement for prediction. If only limited data are available, SWAT requires careful calibration and validation [18]. Here, we applied SWAT with data from one hydrology station and two weather stations to simulate streamflow discharge, assess sediment yield, and perform calibration and validation to help identify areas of high soil loss potential in the Xinjiang River Basin.

The Xinjiang River Basin (27°32′–28°59′ N, 116°38′–118°36′ E) is one of five sub-basins of Poyang Lake Basin, which is situated at the south bank in the middle–lower reach of the Yangtze River in China. Poyang Lake has a large freshwater storage capacity, especially during the summer, and discharges to the Yangtze River. This region is a hotspot of biodiversity and was designated by Wetlands International as a wetland of international importance [19]. However, frequent rainstorms, floods, and subsequent soil erosion have occurred in this basin, with changing climatic conditions and intensive human activity leading to degradation [20–23]. The study area is influenced by a subtropical monsoon climate, with the temporal distribution of rainfall occurring primarily during April–August, thereby driving intense surface land erosion. The Quaternary red soil, with dense clay texture and low permeability, is vulnerable to erosion and widely distributed throughout the basin. Additionally, intense human activity, such as deforestation, mining, and urbanization has accelerated the local soil erosion rate. As a result, in the 2000s, soil loss was estimated at approximately 3.4 × 10^4^ km^2^, accounting for 20.0% of the total land area, and associated with financial losses of up to $333 million [24]. Due to soil loss, the siltation of Poyang Lake is estimated to be 1.2 × 10^7^ ton. This sediment loading and subsequent siltation have dramatically altered the storage of water in Poyang Lake [25].

Soil erosion and lake sediment load issues have increasingly received research attention in the Poyang Lake basin. These studies were roughly categorized into three aspects: (1) The spatiotemporal distribution pattern of long-term precipitation or rainfall erosivity [26–33], (2) the impact of climate change and land use change on soil erosion, runoff, and/or sediment loading based on long-term hydrology and climate observed data [25,34–41], and (3) assessment of soil erosion based on Geographic Information System (GIS), remote sensing, and the universal soil loss equation (USLE) [19,42–45]. These studies resulted in improved understanding of the interactive impact among climate change, hydrologic process, soil erosion, and sediment yield. However, few of the aforementioned studies developed a model to simulate hydrologic patterns and evaluate sediment yield at the required time and spatial resolution to inform land use types (e.g., agriculture) or geographic characteristics (e.g., slope) needed for soil erosion control solutions at the small watershed scale under changing land use practices.

This study predicts long-term streamflow discharge and lake sediment load by applying SWAT in the Xinjiang River Basin and employs calibration and validation steps to generate a model and approach to help identify basin characteristics to support land use and management practice decisions. We set up this SWAT model with limited data and determined the most sensitive hydrologic parameters using the SWAT-CUP and SUFI-2 method [46], which helped to improve calibration and uncertainty analysis. Then, we evaluated the model performance with *R*^2^ and *NSE* in streamflow and sediment prediction and analyzed the uncertainty of the resulting model with *PBIAS*, *RSR*, *p-*factor, and *r-*factor statistics. Finally, we presented high-risk soil loss potential areas within sub-basins. Together, this case study demonstrates an approach and application to help identify the upland sources and magnitude of sediment loads to Poyang Lake from the Xinjiang River Basin and similar systems.

## 2. Materials and Methods

### 2.1. Study Area

**Figure 1.**
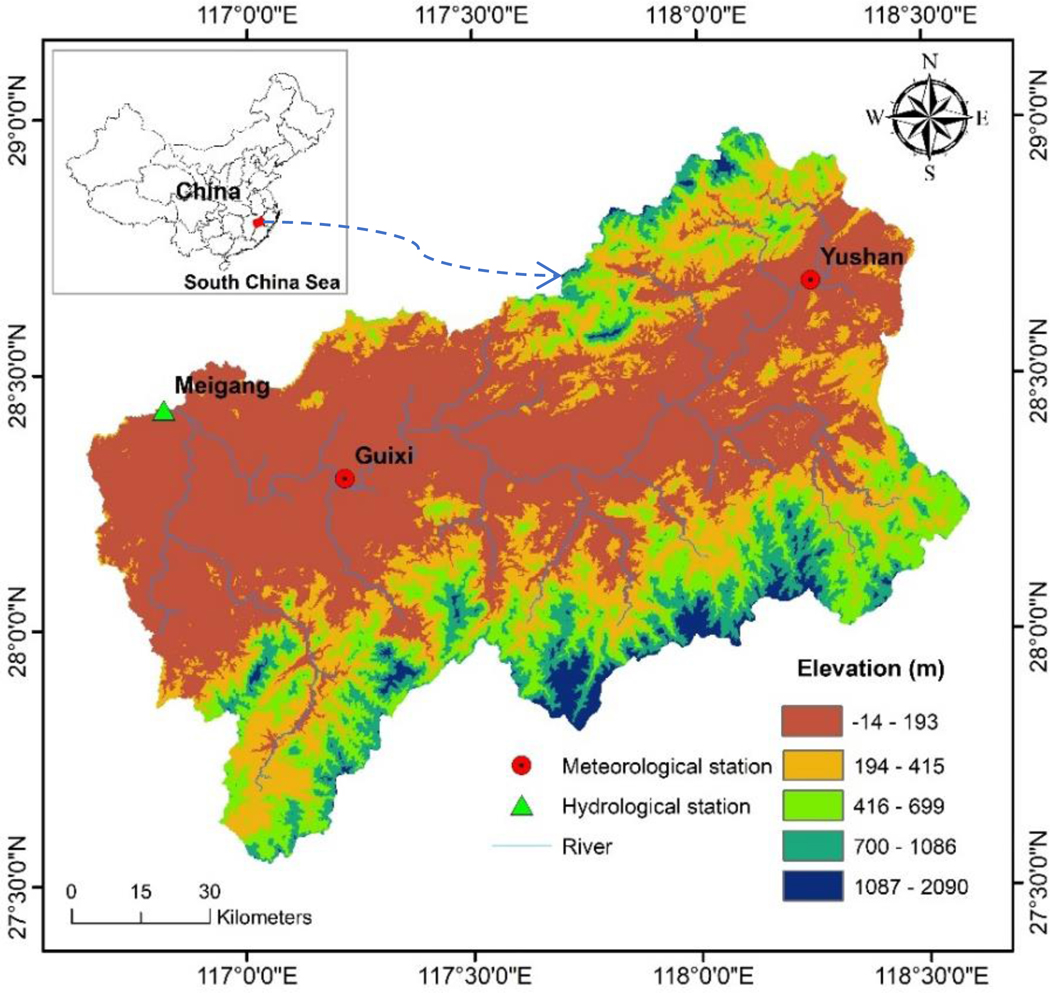
Topography, rivers, and hydrological and meteorological stations of the study area.

**Figure 2.**
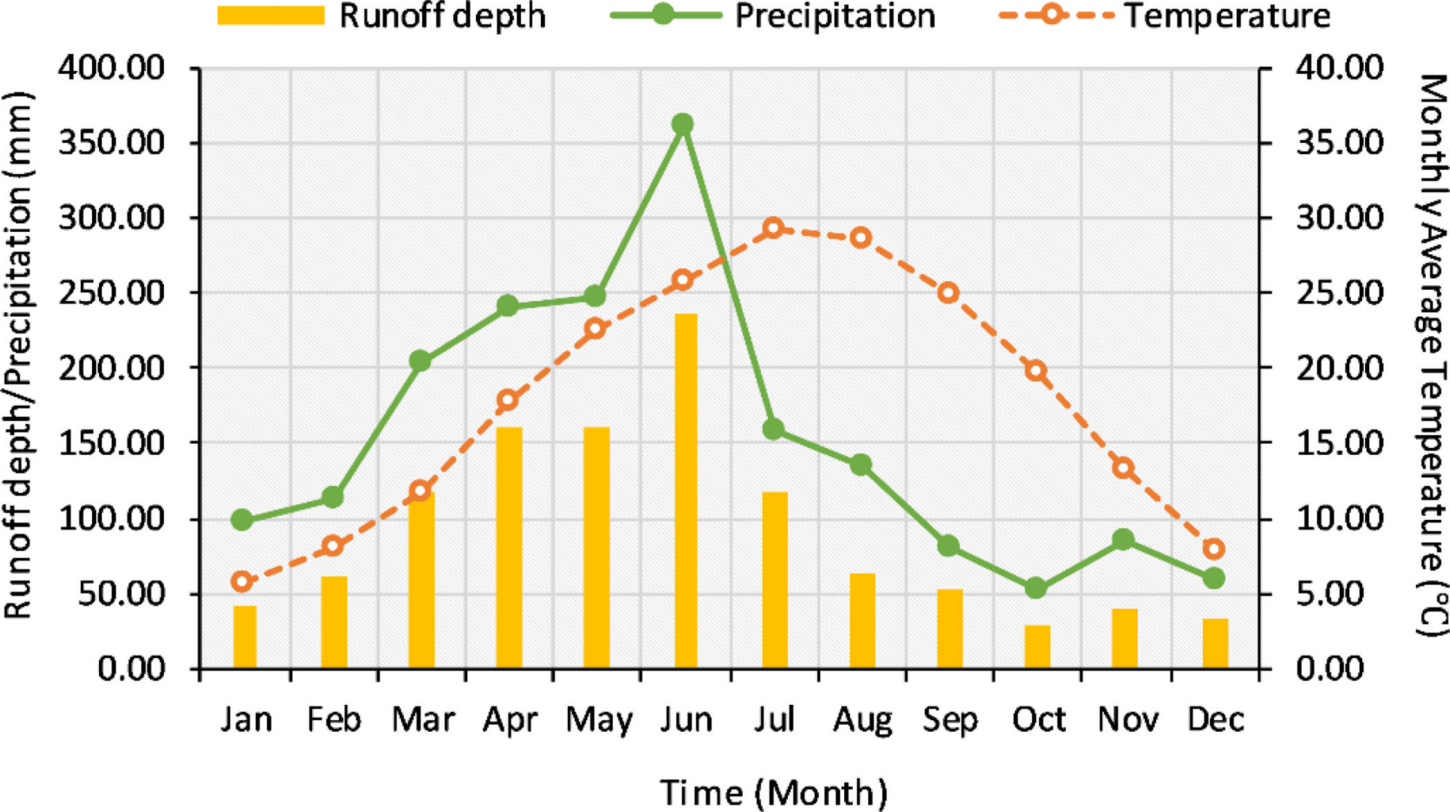
Monthly average runoff depth, precipitation, and temperature during 1985–2009 in the Xinjiang River Basin.

The Xinjiang River Basin (27°32′–28°59′ N, 116°38′–118°36′ E) is located at the eastern part of the Poyang Lake watershed and occupies over 1.7 × 10^5^ km^2^. The southeastern terrain is high in elevation and the northwest is low. The elevation of the Xinjiang ranges from −14 to 2090 m. The length of the main river channel is approximately 360 km. The Xinjiang River flows into Poyang Lake at Meigang station [47]. The upstream area of this station is about 15,535 km^2^. The annual streamflow averaged at Meigang station during 1985–2009 was 555 m^3^ s^−1^. Guixi (28.18° N, 117.13° E, elevation: 50 m) and Yushan (28.41° N, 118.15° E, elevation: 100 m) meteorological stations are located at the lower and upper reaches, respectively (Figure 1). The subtropical monsoon climate system dominates the entire basin. Rainfall increased from January to June, then decreased sharply from July to December [30,48]. The average annual runoff depth, precipitation, and mean temperature values were 1112 mm, 1834 mm, and 18 °C during 1985–2009, respectively (Figure 2).

### 2.2. SWAT Model Description

SWAT is a physically-based and basin-scale model operated at daily or hourly time steps. SWAT evaluates the impact of climate change and land use change on the hydrology cycle in a complex watershed with different soils, vegetation cover, and management conditions [18]. Land use, soil, weather, and topography are the primary input data imported into SWAT. In SWAT, a watershed is discretized into multiple sub-basins, then each sub-basin is further divided into multiple hydrological response units (HRUs). In each HRU, land areas have specific land use, soil property, and slope combinations and hydrological components are calculated for surface water and groundwater [49]. SWAT simulates the hydrologic cycle on a land surface based on the water balance equation (Equation (1)) [49].

(1) $$SW_{t}=SW_{0}+\sum_{n=1}^{t}\left(R_{d}-Q_{surf}-E_{a}-W_{s}-Q_{gw}\right)$$

where *SW*_*t*_ is the final moisture content of soil (mm H_2_O), *SW*_0_ is the initial moisture content of soil on day *i* (mm H_2_O), *t* is time (days), *R*_*d*_ is the total precipitation amount on day *i* (mm H_2_O), *Q*_*surf*_ is the surface runoff amount on day *i* (mm H_2_O), *E*_*a*_ is the evapotranspiration amount on day *i* (mm H_2_O), *W*_*s*_ is the volume of water entering the unsaturated zone from soil profiles on day *i* (mm H_2_O), and *Q*_*gw*_ is the groundwater recharge amount on day *i* (mm H_2_O).

The modified universal soil loss equation (MUSLE) is used in SWAT to calculate a single event sediment yield (Equation (2)) [50].

(2) $$SY=11.8\times {\left(Q_{s}\times q_{p}\times \text{A}\right)}^{0.56}\times K\times LS\times C\times P\times F$$

where *SY* is the sediment yield on a day (ton), *Q*_*s*_ is the runoff volume (mm ha^−1^), *q*_*p*_ is the runoff rate at peak flow (m^3^ s^−1^), *A* is the area of HRU (ha), *K* is the soil erodibility factor, is *LS* the slope length and slope factor, *C* is the vegetation cover factor, *P* is the land management practice factor, and *F* is the coarse fragment factor. The *K*, *C*, *P*, *LS* factors come from USLE.

### 2.3. Input Data

**Figure 3.**
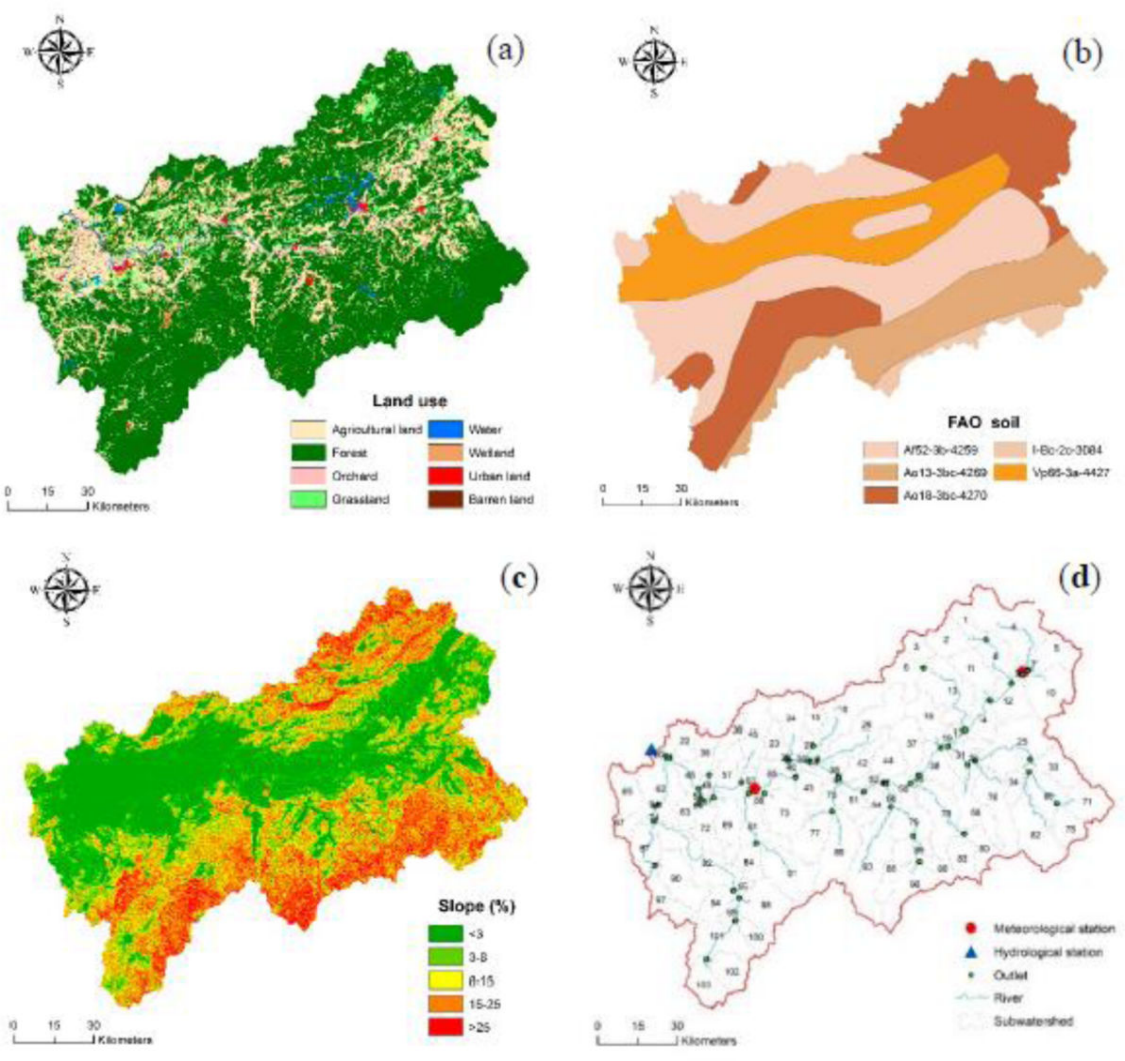
Model data in the Xinjiang River Basin: (**a**) Land use and land cover in 2005; (**b**) Food and Agriculture Organization of the United Nations (FAO) soil; (**c**) slope classifications; (**d**) meteorological and hydrological stations, rivers, outlets, and sub-watersheds.

We obtained the Digital Elevation Model (DEM) of the study area from Shuttle Radar Topography Mission (SRTM) 1 Arc-Second (about 30 m × 30 m) Global Database, which was downloaded from the United States Geological Survey (USGS) (https://earthexplorer.usgs.gov/, 12/20/2017) (Figure 1). Vegetation cover was categorized into agricultural land (24.26%), forest (68.50%), orchard (0.23%), grassland (3.15%), water (1.97%), wetland (0.42%), urban land (1.46%), and barren land (0.01%) (spatial resolution was 100 m × 100 m) (Chinese Academy of Sciences, 2005) (Figure 3a). Due to the unavailability of high-resolution soil data, we downloaded a vectorized soil map (1: 5,000,000) from Food and Agriculture Organization of the United Nations (FAO) soil database (version 3.6) as the SWAT model soil input (http://www.fao.org/geonetwork/srv/en/metadata.show?id=14116, 12/05/2017) (Figure 3b). Table 1 lists the soil ID, name, texture name, composition, and other hydrological properties of each soil type. We used these soil properties to define the basin SWAT soils database. The slopes were divided into five categories to calculate sediment yield from different slopes, including <3%, 3%–8%, 8%–15%, 15%–25%, and >25% (Figure 3c). Daily meteorological data for the period 1985–2009 were obtained from Guixi and Yushan stations. The daily streamflow discharge (1985–2009) and suspended sediment load data (1985–2001) from Meigang gauging station were collected to calibrate and validate the model (Figure 3d).

### 2.4. Model Setup

DEM, FAO soil, and vegetation cover spatial data were converted to a grid raster format, then projected and transformed to the Universal Transverse Mercator (WGS_1984_UTM_Zone_50N) projection coordinate system in the ArcGIS 10.4.1 (Environmental System Research Institue, Redlands, CA, U.S, 2016) desktop environment before setting up the SWAT model. The Xinjiang River basin was delineated into 103 sub-basins with 1094 HRUs. The land use/soil/slope combination defined in HRUs was allocated by land use (10%), soil (10%), and slope (5%) thresholds to produce each response unit. The FAO soil data were appended into a user soil file in the SWAT2012 database before soil definition. The weather parameters obtained from Guixi and Yushan weather stations were written into the WGEN_user file in SWAT2012 database to create a user-defined weather generator. We set a warm-up period of 1985–1989 to stabilize the model in ArcSWAT version 2012.10_4.19 (Texas A&M University, College Station, TX, USA). The simulation running period of the SWAT model was from 1 January 1985 to 31 December 2009.

### 2.5. Model Sensitivity Analysis

The objective of the sensitivity analysis was to scan and find the most sensitive parameters that represented key physical processes [51]. We used global sensitivity analysis methods (or *All-at-a-time, AAT*) to identify the sensitive parameters related to streamflow and sediment yield prediction. The parameter sensitivity was calculated through multiple regression method using the Latin Hypercube (LH), and the objective function values of the parameters were determined by Equation (3) [46].

(3) $$E=a+\sum_{i=1}^{n}\lambda_{i}b_{i}$$

where *E* is the result of an objective function, *a* is a constant of regression analysis, and λ corresponds to the coefficient of each parameter *b*. The significance of parameter *b* was identified using the *t*-test. The sensitivity of one parameter depended on a larger absolute value of the *t-*test and a smaller *p-*value.

### 2.6. Model Calibration, Validation, and Evaluation

We used the SWAT Calibration Uncertainty Procedure (SWAT-CUP) (version 5.1.6.2) (Texas A&M University, College Station, TX, U.S., 2015) and the sequential uncertainty domain parameter fitting algorithm (SUFI-2) integrated into SWAT-CUP to conduct the sensitivity analysis, calibration, validation, evaluation, and uncertainty analysis of the model. After the most sensitive parameters for streamflow and sediment simulation were found, we applied the parameters to conduct the calibration and validation procedures. Due to the limitations of the observed sediment data, we used 1990–1999 as a calibration period of streamflow and sediment and set 2000–2009 and 2000–2001 as the validation periods for streamflow and sediment, respectively.

We evaluated previously published recommendations for calibration and validation [46,52,53], and determined the Nash–Sutcliffe efficiency (*NSE*), Pearson’s coefficient of determination (*R*^2^), percent bias (*PBIAS*), root mean square error (RMSE)-observation’s standard deviation ratio (*RSR*), the *p*-factor, and the *r*-factor as evaluation indicators of the model performance. The values of *R*^2^, *NSE*, *PBIAS,* and *RSR* were calculated using Equations (4)–(7).

(4) $$R^{2}=\frac{{\left(\sum_{i=1}^{n}\left({\text{y}}_{i}-\bar{y}\right)\left({\text{y}}_{i}^{\prime }-\bar{{\text{y}}^{\prime }}\right)\right)}^{2}}{\sum_{i=1}^{n}{\left({\text{y}}_{i}-\bar{y}\right)}^{2}\sum_{i=1}^{n}{\left({\text{y}}_{i}^{\prime }-\bar{{\text{y}}^{\prime }}\right)}^{2}}$$

(5) $$NSE=1-\frac{\sum_{i=1}^{n}{\left({\text{y}}_{i}-{\text{y}}_{i}^{\prime }\right)}^{2}}{\sum_{i=1}^{n}{\left({\text{y}}_{i}-\bar{y}\right)}^{2}}$$

(6) $$PBIAS=\frac{\sum_{i=1}^{n}\left({\text{y}}_{i}-{\text{y}}_{i}^{\prime }\right)*100}{\sum_{i=1}^{n}{\text{y}}_{i}}$$

(7) $$RSR=\frac{\sqrt{\sum_{i=1}^{n}{\left({\text{y}}_{i}-{\text{y}}_{i}^{\prime }\right)}^{2}}}{\sqrt{\sum_{i=1}^{n}{\left({\text{y}}_{i}-\bar{y}\right)}^{2}}}$$

where *y*_*i*_ is the observed data, $\bar{y}$ is the average of the observed data, ${\text{y}}_{i}^{\prime }$ is the predicted data, $\bar{y^{\prime }}$ is the average of the predicted data, *n* and is the sample number. The *p-*factor and *r-*factor described the uncertainty of the simulation. Positive values of *PBIAS* indicated the underestimation bias of the model, and negative values indicated the overestimation bias of the model. An *RSR* value equal to zero indicated a perfect model fit to the measured data, while larger positive *RSR* values indicated a poor model performance [53]. The *p-*factor indicated the percentage of observed data that was captured by the 95% prediction uncertainty (95PPU) band. The *r-*factor represented the thickness of the 95PPU and was estimated to be the average 95PPU thickness divided by the standard deviation of the corresponding observed variable [46].

## 3. Results

### 3.1. Parameter Sensitivity Analysis

**Figure A1.**
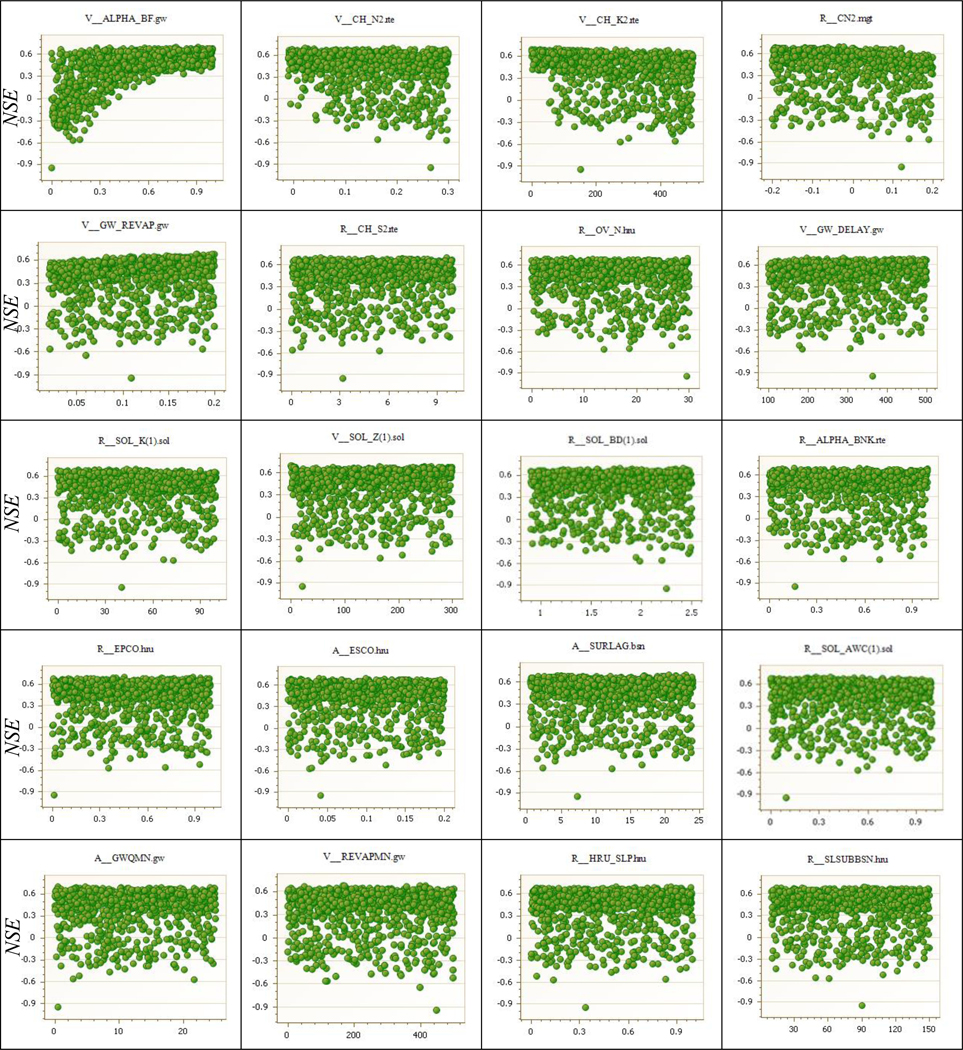
Dot plot of *NSE* against twenty SWAT parameters conditioned with SUFI-2 based on 1000 monthly streamflow calibration simulations. The *x*-axis shows the range of each parameter value and *y*-axis represents *NSE*.

We selected twenty parameters as the initial input for the model sensitivity analysis because these parameters often have high correlation with streamflow simulations, according to the SWAT manual and the literature [18,35,54,55]. We conducted the parameter sensitivity analysis using the *AAT* method based on the SUFI-2 algorithm and LH sampling methods by running 1000 model simulations (see Figure A1 in Appendix A). The final sensitivities of the streamflow parameters were ranked, with the most sensitive parameter given rank 1 and the least sensitive parameter given rank 20, as shown in Table 2.

The sensitivity ranks of each hydrologic parameter applied for the monthly and daily predictions are listed in Table 3. The results revealed that the base flow alpha factor (ALPHA_BF) was the most sensitive parameter for the monthly and daily streamflow simulations, indicating the streamflow simulation in the study area was influenced and dominated by surface runoff and groundwater-flow process.

### 3.2. Streamflow Calibration and Validation

Using the SWAT-CUP, we conducted a one-time iteration of 500 and 1000 model runs in each iteration for the monthly and daily streamflow predictions, until an acceptable accuracy of model simulation was obtained (see Table A1 in Appendix 1) [53]. We used the evaluation guidelines for hydrology simulation from Moriassi and others [53] and Ayle and others [55], which provided recommendations for calibration and validation, and in our limited data case study we showed how this model compared these criteria. We applied 1990–1999 data to calibrate and determine the parameter value ranges, then validated the model with 2000–2009 data. The results of the monthly and daily streamflow calibration and validation statistics are listed in Table 4.

#### 3.2.1. Monthly Streamflow Simulations

**Figure 4.**
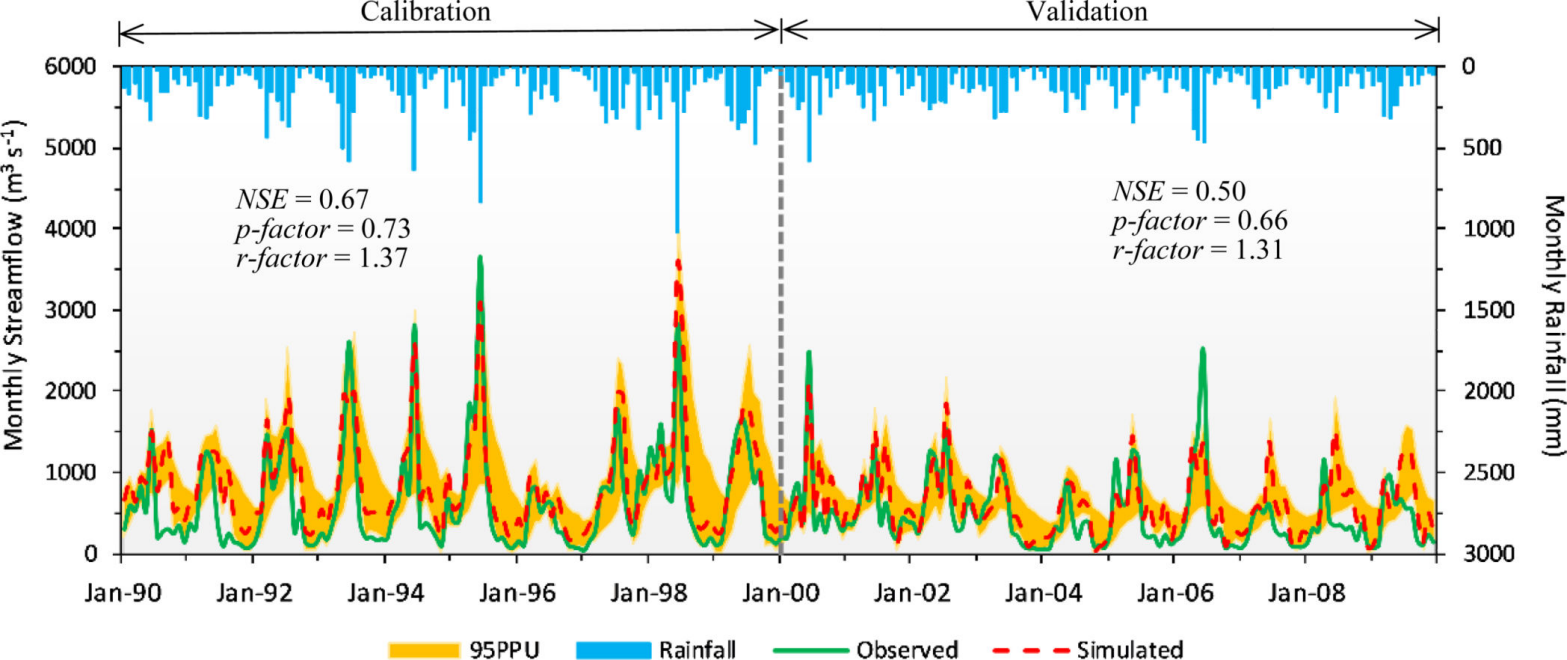
Observed/simulated monthly streamflow hydrograph with 95% predication uncertainty (95PPU) for calibration and validation.

We plotted the hydrograph of the monthly streamflow using the 95% prediction uncertainty (PPU) band and the rainfall bar, and marked the evaluation statistics *NSE*, *p-*factor, and *r-*factor on Figure 4. The results showed that *NSE* was 0.67 in the calibration and 0.50 in the validation. According to Table 4, the monthly streamflow simulation *R*^2^ was 0.79 in the calibration and 0.60 in the validation. The results of *NSE* and *R*^2^ indicated that the observed and simulated monthly streamflows were consistent. The values of *PBIAS* and *RSR* were −33.6% and 0.57 in the calibration and −26.8% and 0.71 in the validation. The *p-*factor value of 73% showed that this percentage of observed data was covered by the 95PPU band in the calibration and 66% of the observed data was captured by the model in the validation, demonstrating that the model simulated the observed data during both time steps. The *r-*factor value was 1.37 in the calibration and 1.31 in the validation, reporting that similar uncertainty occurred in the calibration and validation periods.

#### 3.2.2. Daily Streamflow Simulations

**Figure 5.**
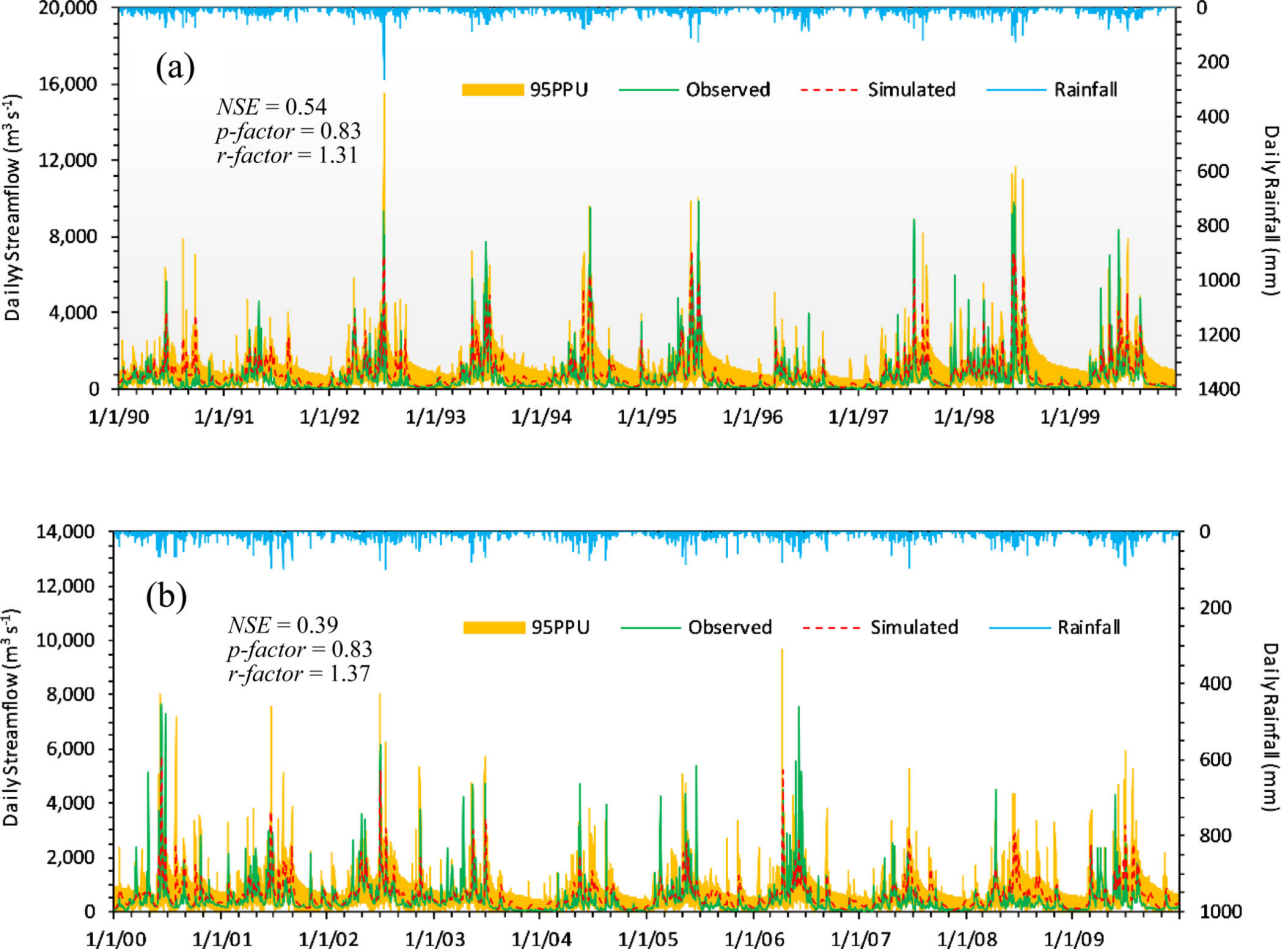
Daily observed streamflow, 95PPU, and best-simulated streamflow. (**a**) Calibration and (**b**) validation.

The *R*^2^ and *NSE* valued for the daily time steps were 0.60 and 0.54 in the calibration and 0.43 and 0.39 in the validation (Table 4). Considering the smaller time scale (daily), we regarded these results as an acceptable agreement between the observations and simulations. To clearly illustrate the simulated result, we plotted Figure 5 to compare the calibrated and validated daily streamflow hydrographs alongside rainfall. Figure 5 shows that the 95PPU predicted 83% of daily observations for both time steps, indicating that the calibrated daily streamflow model simulated daily streamflow in the basin. The simulated daily streamflow was consistent with the daily rainfall as well. However, the *PBIAS* values were −33.1% and −27.7% in the calibration and validation periods, showing that the model overestimated the daily streamflow in both time steps. The model demonstrated proximate uncertainty in calibration and validation (*r*-factor was 1.31 and 1.37), with a relatively large uncertainty at low flow, indicated by a wide 95PPU bandwidth during the lowest discharge. Among the observed flows greater than 4000 m^3^ s^−1^ (total of 7305 daily observations from 1 January 1990 through 31 December 2009, with 109 observations greater than 4000 m^3^ s^−1^), only 28 simulated values were 20% more or less than the observed flows. Although the simulation appeared to capture peak flow well within the 95PPU band, 25.7% (28/109) of the simulated flows fell into the 20% more or less than the observed flow greater than 4000 m^3^ s^−1^ category during the calibration and validation periods.

**Figure 6.**
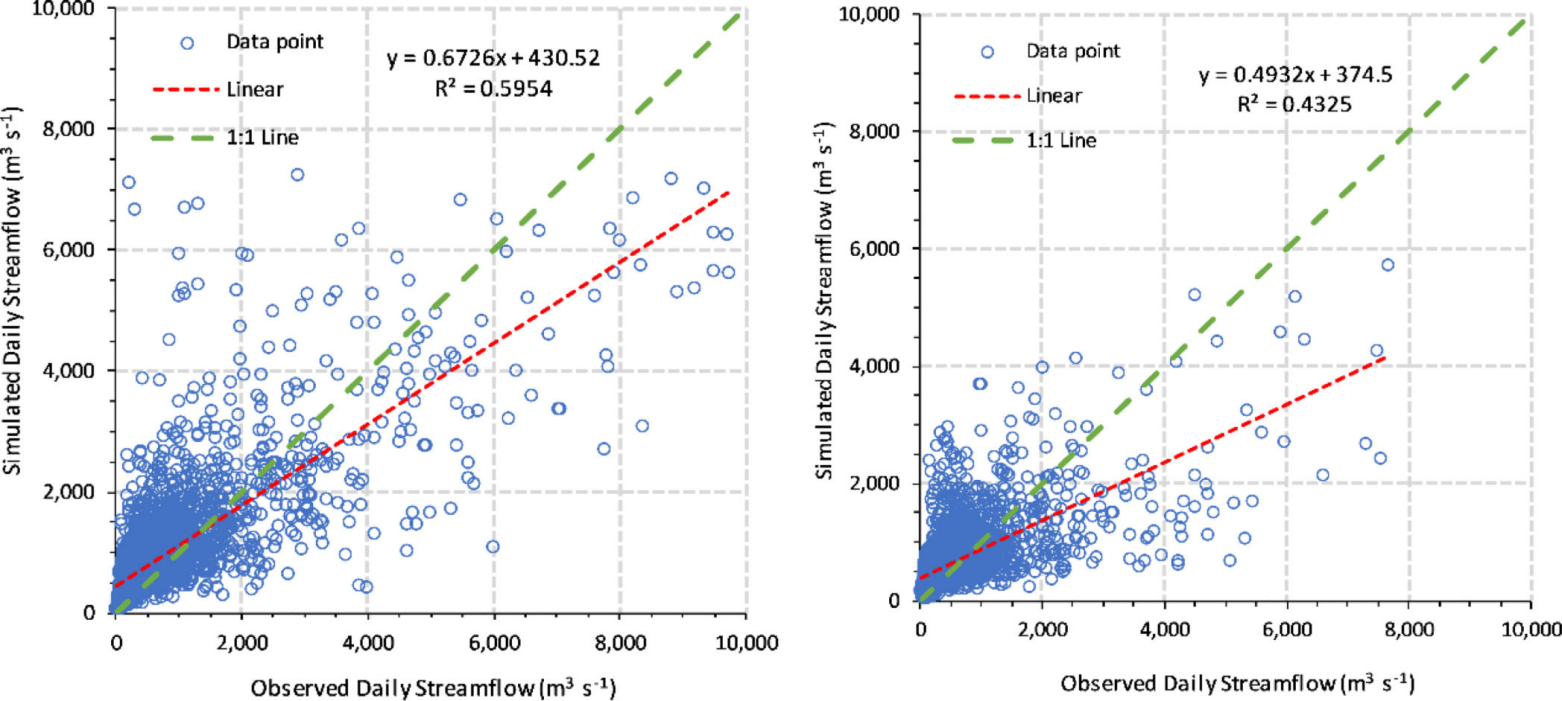
Comparison of observed and simulated daily streamflows. (**a**) Calibration and (**b**) validation.

We illustrated a scatter plot with 1:1 and regression lines to compare the results between the observed and simulated daily streamflows during calibration and validation (Figure 6). The model overpredicted the flow when the observed values were less than approximately 1200 m^3^ s^−1^, yet underestimated flow when the observed value was greater than 1200 m^3^ s^−1^ during calibration (Figure 6a). The model had a large error of prediction when it predicted a daily streamflow peak greater than 4000 m^3^ s^−1^. During validation, the low-flow values (<2000 m^3^ s^−1^) were scattered near the 1:1 line, but most of the high-flow values (>2000 m^3^ s^−1^) were underpredicted (Figure 6b).

### 3.3. Sediment Calibration and Validation

#### 3.3.1. Monthly Sediment Simulations

We appended five control parameters influencing sediment transport simulation to further estimate the suspended sediment load. These sensitive parameters and their details are listed in Table 5. The results showed the linear factor for channel sediment routing (SPCON) was the most sensitive parameter for suspended sediment prediction. Meanwhile, we separated the calibration and validation periods of sediment simulation into 1990–1999 and 2000–2001 to keep the value ranges of the streamflow parameters unchangeable, which were calibrated in the previous sensitivity analysis.

Table 6 shows the overall performance of the monthly sediment simulation for the sediment yield prediction and evaluation under the current conditions with limited data. According to Table 6, a *PBIAS* value of 3.8% indicated that the simulation slightly underestimated the observed suspended sediment in calibration, but a *PBIAS* value of −26.1% in validation indicated that the model overestimated the observed suspended sediment.

**Figure 7.**
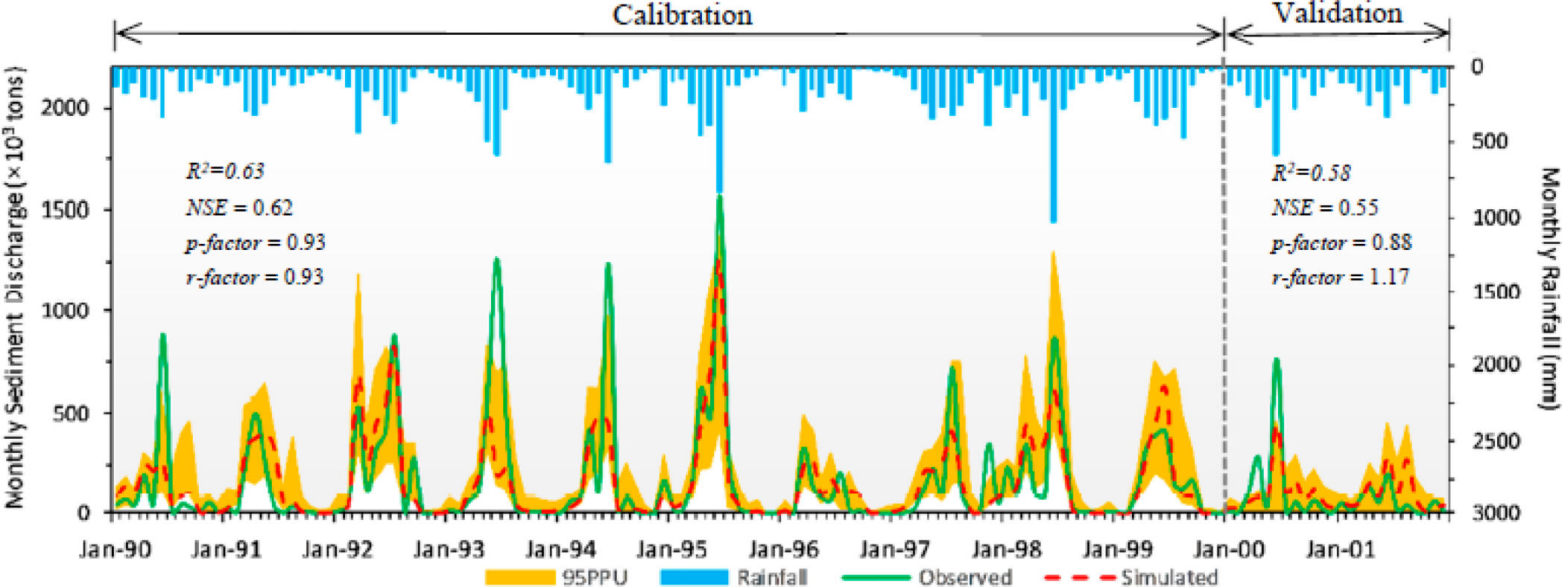
Monthly sediment load for calibration (1990–1999) and validation (2000–2001).

We plotted the sediment graph of the monthly suspended sediment load and marked statistical indicators in Figure 7, with an *R*^2^ of 0.63 and an *NSE* of 0.62 for calibration and an *R*^2^ of 0.58 and an *NSE* of 0.55 for validation. The values of *PBIAS* and *RSR* were 3.8% and 0.61 during calibration and −26.1% and 0.67 during validation, respectively. We used two statistics, *p-*factor and *r-*factor, to present the simulation uncertainty of the sediment prediction by the model. A *p-*factor value of 0.93 in the calibration and 0.88 in the validation indicated that the 95PPU band captured most of the observed data. An *r-*factor value of 0.93 in the calibration and 1.17 in the validation showed a smaller uncertainty of the sediment simulation.

#### 3.3.2. Annual Sediment Simulation

We estimated the annual suspended sediment load (Table 7) throughout the period of simulation (1990–2001). The annual suspended sediment load simulated was 206.2 × 10^4^ ton, which was approximately 13% higher than observed suspended sediment load of 182.3 × 10^4^ ton, with an *R*^2^ value of 0.53 and an *NSE* of 0.40, with the greatest difference occurring under low loads.

Table 8 lists some previous study results regarding the suspended sediment load at Meigang station. Compared with results from previous studies, we believe that the simulated averaged annual sediment yield mass of 206.2 × 10^4^ ton along with observed value of 182.3 × 10^4^ ton are rational and acceptable.

#### 3.3.3. Spatial Variability of Sub-Basins’ Sediment Yield

**Figure 8.**
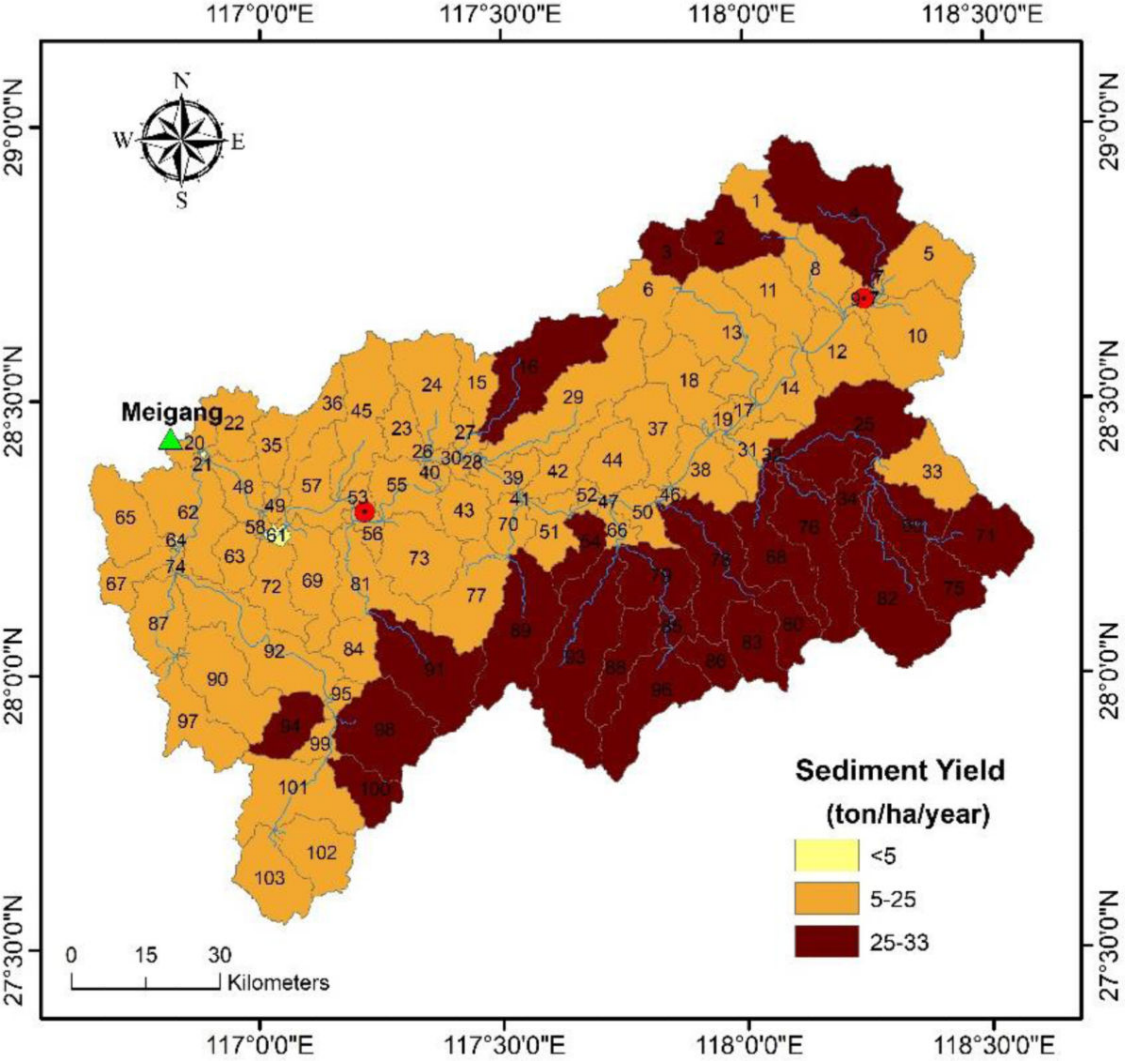
Average annual sediment yield of delineated sub-watersheds in the Xinjiang River Basin (1990–2001).

The annual sediment yield rates varied from 3 ton ha^−1^year^−1^ in the riparian lowlands of the Xinjiang main river channel to 33 ton ha^−1^year^−1^ primarily in the mountain highlands from 103 contributing sub-basins, with an average sediment yield rate of 19 ton ha^−1^year^−1^ for the entire basin (Figure 8). These results were similar in magnitude to the 20.7 ton ha^−1^year^−1^ from Lu et al. [42].

The proportional areas of different sediment yield rate intensities are shown in Table 9. Overall, 99.9% of the study area produced more than 5 ton ha^−1^year^−1^. A total of 60.3% of the land area produced 5–25 ton ha^−1^ year^−1^, and 39.6% of the land area produced 25–33 ton ha^−1^year^−1^. According to our simulation, nearly the entire basin experienced soil loss during 1990–2001.

#### 3.3.4. Sediment Yield Distributed by Slope and Land Use

We determined an average sediment yield of different slope classifications (Table 10) and found that terrain with greater than 25° slopes was a primary sediment source that occupied only 9.5% of total land area, but contributed 24.2% of the entire basin sediment yield. The areas with slopes of 8°– 15° and 15°–25° were also significant sediment source areas that contributed 21.2% and 23.0% of the total basin sediment yield. The disproportionality of sediment yield suggested that soil erosion control measures on slopes greater than 8°, and more so on slopes greater than 25°, may be warranted.

The average annual sediment yield proportions of different land use types are listed in Table 11. Only land uses regarded as sediment “sources” were considered; otherwise, water bodies and wetlands that belonged to sediment “sinks” were not considered in this calculation. Additionally, we did not include urban land, since this was typically an impervious surface. Table 11 shows that orchards were the main contributors of sediment, accounting for 24.1% of the total sediment yield with only 0.2% of the area proportion. Forest was the second most significant contributor to basin sediment, at 21.9% with 68.5% area proportion.

## 4. Discussion

The sensitivity analysis indicated that the base flow alpha factor (ALPHA_BF) was the most sensitive parameter in the monthly and daily streamflow simulations, and the linear factor for channel sediment routing (SPCON) was most sensitive for the monthly sediment prediction. The model had a better performance in the monthly streamflow prediction intervals than daily time steps, and a poorer performance when predicting low-flow events than high-flow events. The monthly streamflow simulation was reported with an *R*^2^ of 0.79 and an *NSE* of 0.6, as shown in Table 4, indicating that the model captured most of the variance in observations. This ability, however, became weak, with an *R*^2^ of 0.60 and an *NSE* of 0.50 during the validation period. The *RSR* value for validation was greater than the calibration value, indicating better model performance during calibration. The *p-*factor (0.73) and *r-*factor values (<1.5) showed desirable certainty for the monthly streamflow calibration and validation, as seen in Table 4. The streamflow peak corresponded with the maximum rainfall. However, the *PBIAS* values of −33.6% in calibration and −26.8% in validation expressed that the model overestimated the monthly streamflow at both time steps, especially during the low-flow period, with considerable uncertainty. There were several potential factors that may have affected the model uncertainty, including input precipitation data quality, particularly limited weather stations locations and the spatial discretization of weather data, coarse soil data input, the Soil Conservation Service (SCS) curve number method itself, unknown processes, and the effect of lumped parameter calculations [46,59]. In this study, we demonstrated that a reasonably well-supported model could be developed with limited data. However, the rainfall data used in our SWAT simulation only came from two meteorological stations, which ultimately limited model performance in a large basin with varied elevation and precipitation patterns. Limited or scarce data would clearly affect uncertainty in future efforts in similar situations, as noted by others (e.g., [55]). Further, the soil data used in our model were derived from FAO, which applies varied approaches to make a best-available determination of relatively coarse soil descriptions (see http://www.fao.org); this may improve over time with enhancements to technology and greater soil data availability.

Guo et al. [35] simulated daily streamflow in the Xinjiang River Basin. The daily streamflow simulation *R*^2^ and *NSE* values were 0.88 and 0.86 in the calibration period (1990–1997) and 0.86 and 0.84 in the validation period (1998–2002) in their study. Obviously, their *R*^2^ and *NSE* values were higher than in our study. However, uncertainty was not reported in their SWAT model and only two statistics were used to estimate the model based on a shorter observed data series. Overall, the model underestimated daily streamflow discharge, as shown in Figure 6, and the model was unable to predict the daily high-flow peak during validation. One possible reason for this is observation error in the high-flow events, because it is difficult to measure high flow, especially during flooding. Overall, our daily SWAT model performed better when simulating daily low streamflow compared with daily high streamflow in this study.

The overall performance of monthly sediment simulation is shown in Table 6 and Figure 7. A time-series comparison of the sediment showed that the observed and simulated suspended sediment load patterns and timing matched well with the rainfall (Figure 7). However, there was a noticeable difference in the monthly sediment simulation time-series and observed values for several high sediment load dates (e.g., June 1990, June 1993, June 1994, July 1997, August 1998, June 1999, and June 2000). High monthly sediment load was not simulated well and was underestimated compared with the corresponding observed data (Figure 7). The likely reason for the error in sediment simulation was the poor hydrologic model simulation of high-flow conditions. Also, the simulated sediment load was higher than the observed sediment in 2001, potentially related to sand-mining activities in the basin beginning in 2001 [22,60].

Although the SWAT model we developed likely underestimated loads in high-flow conditions, our conservative model did provide valuable spatial and geographic insight to landscape drivers of sediment load to the system. Spatially, it was clear that high sediment yield occurred primarily in the highlands, while low sediment yield was mainly found on two banks of the Xinjiang River, with soil erosion particularly severe at the upper reaches of the highlands. Geographically, highland sediment yield of the southern side the Xinjiang main river channel appeared to be a major contributor in the basin (Figure 8). Ayele et al. (2017) [55] showed that the highlands were an important sediment source area, but the sediment ultimately traveled through the lowlands into water bodies. Thus, consideration of both southern highland and lowland practices are important to manage sediment delivery.

According to Huang [56], the area of soil erosion reached 4.1 × 10^3^ km^2^ in 2000, which accounted for 12.3% of the total soil loss area (3.3 × 10^4^ km^2^) in the Poyang Lake basin and was equivalent to 24.7% of the total land area of the Xinjiang River basin. Due to soil erosion, the annual suspended sediment load was 261.1 × 10^4^ ton, accounting for 12.3% of total annual suspended sediment load (2.1 × 10^7^ ton) in the Poyang Lake basin. Meanwhile, Lu et al. (2011) [37] indicated the average depth of topsoil loss in the Xinjiang River basin reached 1.2 mm in 1990; however, this figure increased to 1.5 mm in 2000. This study also showed severe soil erosion during this period.

Since we did not distinguish between dense woodland, sparse woodland, and shrub, these areas were regarded uniformly as forest in our model (Table 11). Forest accounted for the largest area (68.5%) of land use types, which may explain why forest became a primary sediment contribution source in this study. The simplified representation of the forest land use type could have easily resulted in an unexpected simulation output. High resolution land use input may improve future predictions if a SWAT model is used [61–63]. The proportion of barren land was close to zero, but contributed 4.5% sediment yield.

The results of our model-based analysis show that there are land use and geographic hotspots of sediment load. With this insight, several specific management approaches could be considered. Agricultural land occupies 24.3% of the basin but supplies only 17.5% of the sediment yield, suggesting that targeted soil erosion control measures on agricultural land are important, especially on arid farmland distributed on steep southern slopes. Furthermore, poor land tillage practices and deforestation of farmland on steep slopes likely produces more soil erosion and may cause a subsequent increase in sediment yield. Overall, orchards, barren land, and agricultural land are critical sources of sediment yield, while forest and grassland were minor contributors, and dense woodland appeared to contribute a relatively low amount of sediment to the basin.

## 5. Conclusions

Our results showed that the Xinjiang River Basin experienced severe soil loss during 1990–2001, with 99.9% of the total land area contributing a sediment yield rate larger than 5 ton ha^−1^year^−1^. A calibrated and validated SWAT model was able to estimate sediment loads and provide an indication of the land uses and geographic indicators of the primary sources of sediment load. Spatially, the annual sediment yield varied from 3 ton ha^−1^year^−1^ in the lowlands of the two banks of the Xinjiang main river channel to 33 ton ha^−1^year^−1^ in the mountain highlands, with 19 ton ha^−1^year^−1^ on average. Most of the sediment yield came from the southern mountain highlands of the Xinjiang River upstream. Future watershed management should consider the clear influence of land use and slope such as orchards, barren land, and mountain highland on slopes greater than 25°, because these areas tend to be disproportionately large contributors to soil loss and sediment load. Land use management in lowlands could be improved while practicing soil erosion control methods in highlands and inappropriate tillage practices in areas with slopes greater than 25° should be limited to prevent soil loss. Due to our data limitations, we did not compare the impact of land use change on streamflow and sediment load in this watershed, but it is important to determine how the drivers of sediment load change in response to changes in land use, weather, and climate. Nevertheless, this study developed a reliable physically-based streamflow model and illustrated the critical source areas and conditions of sediment yield.

## Figures and Tables

**Table 1.** Soil types and hydrological properties in the Xinjiang River Basin.

Soil Name	Depth of Soil Layer (mm)	Textural Name	Bulk Density (g cm^−3^)	Available Water Capacity (mm mm^−1^)	Saturated Hydraulic Conductivity (mm h^−1^)	Composition		
						Clay (%)	Silt (%)	Sand (%)

I-Bc-2c-	0–300	Clay-	1.0	0.098	38.1	38	33	30
3084	300–550	Loam	1.1	0.098	23.48	41	32	27
Af52–3b-	0–300	Clay	1.2	0.175	14.96	44	23	34
4259	300–1000		1.2	0.175	13.23	52	22	26

Ao13-	0–300	Clay-	1.2	0.129	13.26	39	33	28
3bc-4269	300–670	Loam	1.3	0.129	8.25	45	30	25
Ao18-	0–300	Clay-	1.2	0.144	13.05	37	30	33
3bc-4270	300–770	Loam	1.3	0.144	8.52	44	28	28
Vp66-	0–300	Clay	1.1	0.125	23.2	47	29	25
3a-4427	300–1000		1.3	0.125	8.12	51	25	23

**Table 2.** Calibration streamflow parameters and their adjustable range in the sensitivity analysis.

				Calibration					
No	Parameter Name^1^	Parameter Description	Range	Monthly			Daily		

				*t*-Test	*p*	Rank	*t*-Test	*p*	Rank

1	V__ALPHA_BF.gw	Baseflow alpha factor (1 day^−1^)	0–1	35.76	0.00	1	17.15	0.00	1
2	V__CH_N2.rte	Manning’s “n” value for the main channel	−0.01–0.3	−16.96	0.00	2	−9.16	0.00	2
3	V__CH_K2.rte	Effective hydraulic conductivity in main channel alluvium (mm h^−1^)	−0.01–500	−15.91	0.00	3	−6.19	0.00	3
4	R__CN2.mgt	SCS runoff curve (mm h^−1^)	−0.2−0.2	−7.65	0.00	4	−5.82	0.00	4
5	V__GW_REVAP.gw	Groundwater “revap” coefficient	0.02–0.2	5.31	0.00	5	3.59	0.00	5
6	R__CH_S2.rte	Average slope of main channel (m m^−1^)	−0.001–10	4.26	0.00	6	2.09	0.04	7
7	R__OV_N.hru	Manning’s “n” value for overland flow	0.01–30	−4.21	0.00	7	2.34	0.02	6
8	V__GW_DELAY.gw	Groundwater delay (days)	100–500	2.47	0.01	8	−0.46	0.65	15
9	R__SOL_K (1).sol	Saturated hydraulic conductivity at the 1st soil layer (mm h^−1^)	0–100	−2.04	0.04	9	−1.78	0.08	9
10	V__SOL_Z (1).sol	Depth from soil surface to bottom of the 1st soil layer (mm).	0–300	0.95	0.34	10	0.14	0.89	18
11	R__SOL_BD (1).sol	Moist bulk density at the 1st soil layer (g cm^−3^)	0.9–2.5	−0.90	0.37	11	0.51	0.62	14
12	R__ALPHA_BNK.rte	Baseflow alpha factor for bank storage (days)	0–1	−0.85	0.40	12	−0.46	0.65	16
13	R__EPCO.hru	Plant uptake compensation factor	0–1	−0.59	0.55	13	−0.01	0.99	20
14	A__ESCO.hru	Soil evaporation compensation factor	0–0.2	0.56	0.58	14	0.95	0.34	11
15	A__SURLAG.bsn	Surface runoff lag time	0.05–24	0.55	0.58	15	0.70	0.48	13
16	R__SOL_AWC (1).sol	Available water capacity of the 1st soil layer (mm H_2_O mm soil^−1^)	0–1	0.46	0.64	16	−0.01	0.99	19
17	A__GWQMN.gw	Threshold depth of water in the shallow aquifer required for return flow to occur (mm H_2_O)	0–25	−0.43	0.67	17	1.59	0.11	10
18	V__REVAPMN.gw	Threshold depth of water in the shallow aquifer for “revap” to occur (mm H_2_O)	0–500	0.40	0.69	18	0.94	0.35	12
19	R__HRU_SLP.hru	Average slope steepness (mm^−1^)	0–1	−0.39	0.69	19	0.26	0.80	17
20	R__SLSUBBSN.hru	Average slope length (m)	10–150	−0.23	0.81	20	−1.83	0.07	8

1 Note: “*A*__”, “*V*__” and “*R*__” mean an absolute increase, a replacement, and a relative change to the initial parameter values, respectively. Rank is based on *t-*test and *p-*value [49].

**Table 3.** Calibrated parameters value for the monthly and daily streamflow simulations.

	Monthly			Daily	

Rank	Parameter Name	Value	Rank	Parameter Name	Value

1	V_ALPHA_BF	0.167	1	V_ALPHA_BF	0.700
2	V_CH_N2	0.007	2	V_CH_N2	0.022
3	V_CH_K2	54.491	3	V_CH_K2	482.250
4	R_CN2	−0.175	4	R_CN2	−0.196
5	V_GW_REVAP	0.185	5	V_GW_REVAP	0.176
6	R_CH_S2	8.610	6	R_OV_N	19.039
7	R_OV_N	22.712	7	R_CH_S2	3.294
8	V_GW_DELAY	494.500			

**Table 4.** Monthly and daily streamflow calibration and validation statistics.

Statistical Indicators	Calibration (1990–1999)		Validation (2000–2009)	

	Monthly	Daily	Monthly	Daily

*R*^2^	0.79	0.60	0.60	0.43^#^
*NSE*	0.67	0.54	0.50	0.39* ^#^
PBIAS* ^#^	−33.6%	−33.1%	−26.8%	−27.7
***RSR***	0.57	0.68	0.71^#^	0.78^#^
*p-factor*	0.73	0.83	0.66	0.83
*r-factor*	1.37	1.31	1.31	1.37

* Values are outside of recommendations for Moriassi et al. 2007 [53].

# Value is outside of recommendations for Ayele et al. 2017 [55].

**Table 5.** Result of monthly sensitivity analysis and final calibrated sediment parameters.

Parameter	Range	Rank	*t*-Test	*p*-Value	Value

v_SPCON.bsn	0.0001–0.01	1	3.82	0.00	0.005
v_USLE_P.mgt	0–1	2	2.58	0.01	0.522
r_CH_EROD.rte	0–1	3	−0.49	0.63	0.602
v_SPEXP.bsn	1–1.5	4	−0.15	0.88	1.281
r_CH_COV.rte	−0.001–1	5	−0.06	0.95	0.369

**Table 6.** Monthly observed and simulated sediment calibration (1990–1999) and validation (2000– 2001) model performance statistics.

Component	*p-*Factor	*r-*Factor	*R*^2^	*NSE*	*PBIAS*	*RSR*

Calibration	0.93	0.93	0.63	0.62	3.8%	0.61
Validation	0.88	1.17	0.58	0.55	−26.1%	0.67

**Table 7.** Annual observed and simulated suspended sediment loads at Meigang station.

Year	Sediment Load (× 10^4^ ton)		Year	Sediment Load (× 10^4^ ton)	
	Observed	Simulated		Observed	Simulated

**1990**	143.4	196.9	1996	87.0	144.9
**1991**	118.0	186.2	1997	175.5	172.2
**1992**	264.7	242.8	1998	221.7	308.2
**1993**	257.9	213.8	1999	174.9	218.4
**1994**	223.5	213.4	2000	137.3	155.8
**1995**	317.8	251.3	2001	66.2	170.6
**Average**				182.3	206.2

**Table 8.** Observed suspended sediment load at Meigang station.

Authors	Periods	Observed Sediment (× 10^4^ ton year^−1^)

**Guo et al.** [41]	1991–2001	200
**Huang** [56]	-	261
**Sun et al.** [34]	1956–2005	210
**Min et al.** [57]	1956–2005	212
**Luo et al.** [58]	1956–2008	204

**Table 9.** Simulated sediment yield rate categories and proportional areas.

Sediment Yield (ton ha^−1^ year^−1^)	Area (km^2^)	Total Land Area %

<5	20.39	0.1%
5–25	9121.50	60.3%
25–33	5992.87	39.6%

**Table 10.** Simulated sediment yield proportions.

Slope (°)	Area (km^2^)	Area %	Sediment Yield %

<3	4996.8	33.0%	13.3%

3–8	3116.3	21.6%	18.3%
8–15	2871.1	19.0%	21.2%
15–25	2712.9	17.9%	23.0%
>25	1437.6	9.5%	24.2%

**Table 11.** Simulated average annual sediment yield from different mainland use.

Land Use	Area (km^2^)	Area %	Sediment Yield %

Agricultural land	3670.1	24.3%	17.5%
Forest	10,363.5	68.5%	21.9%
Orchard	35.4	0.2%	24.1%
Grassland	477.3	3.2%	16.7%
Barren land	1.3	0%	4.5%

## Appendix A

Parameter sensitivity analysis is an important step to effectively limit the number of parameters and identify key parameters [64]. The *AAT* method was applied to search optimize parameters. A total of 1000 model runs were used to identify sensitive parameters in this study. The final sensitivities are ranked in Table 2. There is potential for the order of parameter sensitivity to differ if the number of runs changes.

Figure A1 is based on 1000 SUFI-2 monthly simulations. The *x* axis shows the range of parameter values for streamflow and the *y* axis is the value of the objective function, *NSE*. If the distribution of these dots showed an obvious trend, the sensitivity would be higher; if the points were scattered or haphazard, the parameter sensitivity would be low. ALPHA_BF, CH_N2, CH_K2, CN2, and GW_REVAP were the most sensitive parameters in the streamflow simulations of the study area (Figure A1).

Abbaspour et al. (2015a) determined that a *p*-value of <0.05 was the threshold for a sensitive parameter. Although the *p*-value of SOL_K (1) was 0.04, other relevant soil parameters, such as SOL_Z (1), SOL_BD (1), and SOL_AWC (1), were insensitive in analysis. Moreover, since the soil data came from the FAO soil database constructed from filed surveys backed up by remote sensing, environmental data, expert opinions, and laboratory analyses, the values of these soil property parameters were considered subject to less uncertainty and thus were not included in the final calibration process. ALPHA_BF was the baseflow alpha factor (1 day^−1^), reflecting the baseflow response to changes in the recharge of the shallow aquifer. The larger the value, the more sensitive the baseflow was to recharge. CH_N2, the Manning’s “n” value for the main channel, defined the channel flow resistance. CH_K2 referred to the effective hydraulic conductivity in the main channel alluvium (mm h^−1^), and showed stream-groundwater relationships. CN2 was a key parameter in the SCS method of SWAT, characterizing the potential maximum soil moisture retention capacity. A low value indicated low runoff, but high potential infiltration. GW_REVAP, the groundwater “revap” coefficient, was the rate of the water returning to the soil zone from the aquifer when the overlying soil became unsaturated. CH_S2 was the average slope of main channel along the channel length (m m^−1^). OV_N indicated the Manning’s “n” value for the overland flow, and was relevant to the calculation of the time of concentration for overland flow; therefore, this value had an impact on the timing of the flow peaks. GW_DELAY characterized the delay time (days) of the recharge into the aquifer in the unsaturated non-root zone.

As a result, we selected out most sensitive parameters based on the result of the above sensitivity analysis for the Xinjiang River Basin streamflow simulation. Table 4 lists the calibration results of the most influential parameters.

Moriasi et al. (2007) proposed a general guideline for the recommended statistics for the monthly streamflow and sediment simulations (Table A1).

**Table A1.** Performance ratings of recommended statistics for streamflow simulations (modified from [53–55]).

Performance Rating	*R*^*2*^	*NSE*	*RSR*	*PBIAS* (%)	
				Streamflow	Sediment

Very Good	0.7 <*R*^2^ ≤1	0.75 < *NSE* ≤ 1	0 ≤*RSR* ≤0.5	*PBIAS* < ±10	*PBIAS* < ± 15
Good	0.6 < *R*^2^ ≤ 0.7	0.65 < *NSE* ≤ 0.75	0.5 < *RSR* ≤ 0.6	±10 ≤ *PBIAS* < ±15	±15 ≤ *PBIAS* < ±30
Satisfactory	0.5 < *R*^2^ ≤ 0.6	0.5 < *NSE* ≤ 0.65	0.6 < *RSR* ≤ 0.7	±15 ≤ *PBIAS* < ±25	±30 ≤ *PBIAS* < ±55
Unsatisfactory	*R*^2^ ≤ 0.5	*NSE* < 0.5	*RSR* > 0.7	*PBIAS* ≥ ±25	*PBIAS* ≥ ±55
